# The Effects of Vasodilation Induced by Brachial Plexus Block on the Development of Postoperative Thrombosis of the Arteriovenous Access in Patients with End-Stage Renal Disease: A Retrospective Study

**DOI:** 10.3390/ijerph192215158

**Published:** 2022-11-17

**Authors:** Jonghae Kim, Kihyuk Park, Youngjin Cho, Jaehoon Lee

**Affiliations:** 1 Department of Anesthesiology and Pain Medicine, Daegu Catholic University Medical Center, Daegu Catholic University School of Medicine, 33, Duryugongwon-ro 17-gil, Nam-gu, Daegu 42472, Republic of Korea; 2 Division of Vascular and Endovascular Surgery, Department of Surgery, Daegu Catholic University Medical Center, Daegu Catholic University School of Medicine, 33, Duryugongwon-ro 17-gil, Nam-gu, Daegu 42472, Republic of Korea

**Keywords:** arteriovenous access, brachial plexus block, coronary artery disease, diabetes mellitus, renal replacement therapy, vasodilation

## Abstract

Although brachial plexus block (BPB)-induced vasodilation reduces the incidence of arteriovenous access (AC) thrombosis, BPB cannot completely prevent its development. Therefore, we retrospectively investigated the factors affecting BPB-induced vasodilation and their effects on AC thrombosis development. Ninety-five patients undergoing AC surgery under BPB were analyzed. Vessel diameters were measured before and 20 min after BPB. The surgery abandoned before the BPB placement was performed when the BPB-induced increases in vessel diameters met its indications. Complete occlusive access thrombosis (COAT) was defined as loss of pulse, thrill, or bruit. Fourteen patients (14.7%) developed COAT. The outflow vein was more dilated by BPB than the inflow artery (0.6 versus 0.1 mm in median, *p* < 0.001). The original surgery plan was changed for seven patients (7.4%). Diabetes mellitus (DM) and ischemic heart disease (IHD) decreased the extent of increases in the inflow artery by −0.183 mm (95% confidence interval [CI] [−0.301, −0.065], *p* = 0.003) and outflow vein diameters by −0.402 mm (95% CI [−0.781, −0.024], *p* = 0.038), respectively. However, DM, IHD, and changes in the vessel diameters had insignificant effects on the development of COAT. In conclusion, although DM and IHD attenuate the vasodilating effects of BPB, they do not contribute to the development of COAT.

## 1. Introduction

The number of patients with end-stage renal disease in Korea is steadily increasing due to increases in both the incidence of adult diseases such as type 2 diabetes and hypertension and the aging population. The aging population has increased because of medical advances. Dialysis is a renal replacement therapy for patients with end-stage renal disease. It is a popular treatment that can be maintained for several decades or more in place of renal function. At the end of 2019, the number of peritoneal dialysis patients was 6000, which has remained unchanged for 10 years; moreover the number of hemodialysis patients has increased annually, accounting for 93.2% (15,587) of all dialysis patients in Korea [1]. For long-term hemodialysis, arteriovenous access surgery is essential.

Arteriovenous access surgery can be performed under local, regional, or general anesthesia. Administration of general anesthetics to patients with end-stage renal disease increases the risk of developing acute cerebrovascular and cardiovascular events [2]. Although local anesthesia can avoid the risks related to general anesthesia, it causes vasospasms that lead to blood flow impairment and local edema and increases the patient’s risk of early thrombosis and infection of the arteriovenous access [3]. Therefore, brachial plexus block (BPB) as regional anesthesia is more favored than the other two types of anesthesia because its sympathectomy effects cause vasodilation [4] that lasts into the early postoperative period [5,6] and does not cause side effects or complications associated with the administration of local or general anesthesia.

Accordingly, the rate of primary patency (the presence of a thrill or bruit) was higher at 3 months after surgery in patients with BPB than in patients with local anesthesia (84% vs. 62%) [7]. However, BPB could not completely prevent the loss of patency. Nevertheless, the factors that compromise the vasodilating effects of BPB and cause loss of patency have not been investigated in patients undergoing arteriovenous access surgery under BPB.

In this study, we retrospectively investigated the factors affecting the changes in arterial and venous diameters of the upper extremities after the placement of a BPB in patients undergoing arteriovenous access surgery under BPB. Subsequently, the role of the identified factors in the development of arteriovenous access thrombosis was examined.

## 2. Materials and Methods

### 2.1. Participants

The protocol of this retrospective study was approved by the Institutional Review Board of our institution (CR-20-056). This study was exempted from the requirement for written informed consent owing to its retrospective nature. The medical records of 95 patients aged >18 years who underwent primary arteriovenous access surgery under ultrasound-guided BPB between December 2018 and February 2020 were reviewed. Patients who received general or local anesthesia because of contraindications to BPB or failure of BPB were not included in the analysis. The cases of failed surgical procedures were also excluded. The arteriovenous access surgery was performed by one vascular surgeon and anesthetics were administered by one anesthesiologist.

### 2.2. Patient Monitoring and Indications for Brachial Plexus Blocks

The patients fasted from midnight, and an infusion of Plasmalyte was started 1 h before surgery via an intravenous catheter placed into the lumen of the basilic or cephalic vein contralateral to the side of the surgery. Upon arrival to the operating room, the patient was placed on the operating table in the supine position and then acclimatized for 10 min following electrocardiogram, pulse oximetry, and noninvasive blood pressure monitoring. After the baseline vessel diameters were measured, BPB was performed. According to the site of surgery and the patients’ medical conditions, one of the 3 BPB approaches (axillary, supraclavicular, and infraclavicular) was chosen by the anesthesiologist [8]. Axillary BPB was indicated if the surgery involved only the forearm or wrist. If the elbow or upper arm was involved, the patient received supraclavicular BPB. However, patients with severe pulmonary disease, contralateral hemidiaphragmatic paresis or paralysis, or contralateral vocal cord palsy did not receive a supraclavicular BPB because its side effects, such as ipsilateral phrenic or recurrent laryngeal nerve block, could cause respiratory failure. Alternatively, an infraclavicular BPB was placed for patients contraindicated for a supraclavicular BPB.

### 2.3. Brachial Plexus Block

For an axillary BPB, the patient’s axilla was exposed by abducting the upper arm at 90 degrees with the forearm flexed to 90 degrees. A 5-to-13 MHz linear ultrasound transducer (UST-5411, Hitachi Aloka Medical, Ltd., Tokyo, Japan) connected to an ultrasound machine (ProSound α7 Premier, Hitachi Aloka Medical, Ltd.) was placed in the axillary fossa at the intersection of the deltoid and biceps muscles to visualize the median, ulnar, and radial nerves surrounding the pulsating axillary artery and the musculocutaneous nerve within the coracobrachialis muscle or between the biceps and coracobrachialis muscles. For supraclavicular and infraclavicular BPBs, the patient’s head was slightly rotated contralateral to the block side, and the arm was adducted with the hand on the abdomen. For a supraclavicular BPB, the compactly arranged brachial plexus was visualized lateral to the pulsating subclavian artery above the first rib by placing the linear ultrasound transducer in the supraclavicular fossa. During an infraclavicular BPB, the linear ultrasound transducer was placed in a sagittal plane caudal to the distal third of the clavicle and medial to the coracoid process to visualize the lateral, medial, and posterior cords surrounding the pulsating subclavian (axillary) artery below the pectoralis minor muscle fascia.

A 50-mm, 22-gauge, blunt-tipped, echogenic needle (Stimuplex^®^ HNS 12, B. Braun, Melsungen, Germany) was inserted close to the target nerves in the lateral-to-medial (for axillary and supraclavicular BPBs) or cephalad-to-caudal direction (for an infraclavicular BPB) using an in-plane technique. Once the needle tip was placed near the target nerves, an assistant injected 1.5% mepivacaine in 1 mL increments through an injection tube. If significant resistance was encountered or pain/paresthesia was elicited during local anesthetic injection, the needle was withdrawn and then redirected under the suspicion of intrafascicular injection. Although a total of 25 mL of 1.5% mepivacaine was planned to be used, increases of up to 40 mL in the local anesthetic volume were allowed in case 25 mL was not sufficient to block all the target nerves.

### 2.4. Measurement of Vessel Diameters

The vessel diameters were measured 10 min after acclimation and 20 min after BPB placement in the operating room, where the ambient temperature and relative humidity were 21–24 °C and 40–60%, respectively. In the supine position, the patient’s arm was adducted to the body with the elbow fully extended and the forearm supinated. The patient was instructed not to move the evaluated upper limb or the body. The linear ultrasound transducer (UST-5411) was placed perpendicular to the skin to obtain the optimal cross-sectional imaging of the vessels. By freezing the B-mode image and subsequently rolling the trackball to scroll the stored ultrasound images, the best image for the vessel diameter measurement was acquired. An electronic caliper with an accuracy of 0.1 mm, which was located inside the ultrasound machine, was used to measure the vessel diameters. The diameter of a vessel was measured as the maximum distance between the 2 points of a vascular inner wall. Specifically, the diameter of an artery was obtained at the end-diastole of the cardiac cycle. When placing the linear ultrasound transducer for the measurement of the venous diameter, minimal pressure was applied on the skin overlying the vein of interest to avoid compressing the vein and maintain its circular shape. The measurements were taken without a tourniquet. The skin area of the baseline measurement was marked with a skin marking pen to allow subsequent measurement after BPB at the same skin area as the first measurement. The diameters of the radial artery and the forearm cephalic vein were measured at the wrist; those of the brachial artery and the upper arm cephalic vein were measured at the antecubital fossa; and those of the axillary artery and vein were measured at the axilla fossa. One vascular surgeon, who was not involved in the anesthesia and surgery, measured the vessel diameters.

### 2.5. Indications for Arteriovenous Access

Radial-cephalic arteriovenous fistula (AVF) was indicated if the diameter of the radial artery was >2 mm without any calcification inside its lumen and the diameter of the forearm cephalic vein was >2.5 mm with a continuous length >5 cm. Brachiocephalic AVF was performed if the abovementioned indications for radial-cephalic AVF were not fulfilled and the diameter of the upper arm cephalic vein was >2.5 mm. If the whole cephalic vein was unsuitable for AVF, arteriovenous graft (AVG) creation was planned. If the diameter of the antecubital vein was >3 mm, a prosthetic brachial-antecubital forearm loop access was created. If the diameters of the antecubital and basilic veins were ≤3 mm and >3 mm, respectively, a prosthetic brachial-basilic forearm loop access was created. However, if those 2 veins did not have diameters >3 mm, brachial-axillary bridge grafting was performed. An axillo-axillary loop graft was considered as a last resort if no vein was available (≤3 mm) below the axillary vein and the diameter of the brachial artery was ≤3 mm in the presence of diffuse calcification within its lumen. The preset surgery plan was changed with the BPB-induced increases in vessel diameters that met the indications of the surgery abandoned before the placement of BPB.

### 2.6. Measurement of Sensory and Motor Blockade

Following the measurements of vessel diameters, we assessed the sensory and motor blockades of the musculocutaneous, axillary, radial, median, and ulnar nerves. Using an alcohol swab, the loss of cold sensation was determined in the sensory areas of each nerve. The movement force of the muscles innervated by each nerve was also evaluated. However, adequate surgical anesthesia was complete sensory blockade only in the surgical field regardless of the blockade of the area other than the surgical field.

### 2.7. Intraoperative Anesthetic Management

Oxygen was administered via a nasal cannula at a rate of 2 L/min during the surgery. By sampling expired carbon dioxide through the accessory tube from the nasal cannula, which was connected to the capnometer, end-tidal carbon dioxide and respiratory rate were monitored. Noninvasive arterial blood pressure was measured at 5 min intervals, but the measurements were repeated within the intervals when the patient’s medical conditions required more frequent measurements. Blood pressure was maintained within 20% of the baseline value using the medications chosen at the discretion of the attending anesthesiologist.

### 2.8. Postoperative Follow-Up

On postoperative days 1 and 14, we confirmed the patency of the arteriovenous access by assessing the presence of the pulse, bruit, and thrill under palpation and auscultation. At the 6th postoperative week, the flow volume and diameter of the outflow vein were evaluated under ultrasound. If the venous flow volume was >600 mL/min and the venous diameter was >6 mm, the arteriovenous access was determined to be adequately functional, allowing cannulation for hemodialysis.

### 2.9. Outcome Variables

The duration of patency was defined as the time between arteriovenous access creation and the development of complete occlusive access thrombosis followed by thrombectomy or abandonment of the access [9]. Complete occlusive access thrombosis was defined as loss of pulse, thrill, or bruit under palpation and auscultation throughout cardiac cycles (systole and diastole) at least 8 cm proximal to the arteriovenous anastomosis [10] (before any intervention to restore patency). The follow-up was discontinued at the development of complete occlusive access thrombosis. Patients who developed complete occlusive access thrombosis were assigned to the occlusion group, and the other patients were assigned to the patency group. We collected the patients’ demographic data, including age, sex, height, weight, and underlying diseases; BPB data, including the side of surgery, BPB type, block performance time, the volume of local anesthetic used, and duration of anesthesia; surgery data, including surgery type, whether AVF or AVG was performed, whether the original surgery plan was changed, and duration of surgery; and vessel diameters before and after BPB. Block performance time was defined as the time in min between insertion and removal of a block needle during BPB.

### 2.10. Statistical Analysis

Based on the results of the normality test (Shapiro–Wilk test), normally or nonnormally distributed continuous variables are presented as the mean ± SD or median (1st quartile, 3rd quartile), respectively. Categorical data are presented as the number of patients (percentage). Normally distributed data were compared between the 2 groups using an independent 2-sample Student’s *t* test, while nonnormally distributed data were compared using the Mann–Whitney U test. The median difference with 95% CI was calculated using the Hodges–Lehmann estimator. Categorical data were compared between the 2 groups using a chi-square test. However, Fisher’s exact test was performed when the expected frequency of any cell from a 2-by-2 table was less than 5. The Wilcoxon signed-rank test was used to assess the longitudinal changes in vessel diameters from before to after BPB and the within-group difference between vessel diameter changes in both the inflow artery and outflow vein of the arteriovenous access after BPB. Multiple linear regression was performed to assess the effects of independent variables on post-BPB changes in the diameters of the inflow artery and outflow vein. The independent variables included age, sex, body mass index, BPB type, the volume of mepivacaine used for BPB, smoking history, and underlying diseases. Independent variables with a variance inflation factor >5 were regarded as multicollinear with some of the other variables [11]. Independent variables, the variance decomposition proportions of which were >0.3 at the common condition index (>10), were regarded as multicollinear with one another [11,12]. Among the multicollinear variables, more clinically irrelevant variables were excluded first from the regression models. The final multiple linear regression models had differences <0.1 between the coefficient of determination (R^2^) and the adjusted R^2^. We performed univariate Cox proportional hazards regression analysis to predict the occurrence of complete occlusive access thrombosis at a specific time using an independent variable (age, diabetes mellitus, ischemic heart disease, post-BPB changes in the diameters of the inflow artery and the outflow vein from the arteriovenous access, and arteriovenous access type [AVG vs. AVF creation]). However, multiple Cox proportional hazard regression analysis, which requires 10 events per independent variable to obtain an unbiased regression model [13], could not be used because the observed number of events (complete occlusive access thrombosis) in our study was 14, allowing the use of only one independent variable. The effects of diabetes mellitus, ischemic heart disease, and arteriovenous access type on the development of complete occlusive access thrombosis were assessed with Kaplan–Meier survival analysis. The median survival time, restricted mean survival time, and restricted mean time lost were calculated from the survival functions. The Kaplan–Meier survival plots were compared between the 2 groups using a log-rank test. All tests were 2-sided, and *p* < 0.05 was considered statistically significant. Statistical analyses were performed using NCSS 2021 Statistical Software (NCSS, LLC, Kaysville, UT, USA) and IBM SPSS software version 25.0.0 (IBM, Armonk, NY, USA). The purposes of using each statistical method were comprehensively presented in Table S1.

## 3. Results

### 3.1. Patient Characteristics

Out of the 95 patients undergoing arteriovenous access surgery, 14 patients (14.7%) developed complete occlusive access thrombosis, among whom 3 patients had immature AVFs and 6 patients had brachial-axillary bridge grafts (Table 1). No patient required general anesthesia or supplemental local anesthetic infiltration due to failure of BPB. The block performance time for BPB was 5 min in median in the two groups. The percentage of patients receiving AVGs was higher in the occlusion group than in the patency group (78.6% versus 40.7%). There was a significant difference in the number of patients receiving AVFs/AVGs between the two groups (χ [1, n = 95] = 6.871, *p* = 0.009). By benefiting from BPB-induced vasodilation, six patients who were originally planned to receive AVGs received brachial-cephalic AVFs, and one patient originally planned to receive a brachial-cephalic AVF received a radial-cephalic AVF. The patency group was followed-up longer than the occlusion group (*p* = 0.018) because the follow-up was terminated if complete occlusive access thrombosis occurred. No patient developed local anesthetic systemic toxicity.

### 3.2. Changes in Vessel Diameters after Placement of Brachial Plexus Block

BPB significantly increased the diameters of the radial and brachial arteries in the patency group (Table 2). However, no significant increases in the diameters of the two arteries were found in the occlusion group. BPB had no effect on the diameter of the axillary artery in the two groups. BPB significantly increased the diameters of all three veins (the forearm cephalic, upper arm cephalic, and axillary veins) in the two groups. The diameters of the inflow artery and the outflow vein used for the arteriovenous access were significantly increased from baseline after BPB placement in the two groups. The extent of the changes in the diameter of the outflow vein was significantly greater than that of the inflow artery in the two groups. However, no significant differences in the vessel diameters before and after BPB placement or the changes in vessel diameters before and after BPB placement were observed between the two groups.

### 3.3. Factors Contributing to Changes in Inflow Artery and Outflow Vein Diameters of Arteriovenous Access after Placement of Brachial Plexus Block

Diabetes mellitus was found to decrease the extent of the increase in the inflow artery diameter of the arteriovenous access after BPB placement by 0.183 mm (95% CI [−0.301, −0.065], *p* = 0.003) according to the multiple linear regression model after controlling for age, smoking history, ischemic heart disease, and cerebrovascular accident (Table 3). However, diabetes mellitus did not have an effect on the diameter of the outflow vein for the arteriovenous access (Table 4). Ischemic heart disease decreased the extent of the increase in the outflow vein diameter of the arteriovenous access after BPB placement by 0.402 mm (95% CI [−0.781, −0.024], *p* = 0.038) according to the multiple linear regression model after controlling for age, smoking history, diabetes, and cerebrovascular accident.

### 3.4. Effects of Changes in Inflow and Outflow Vessel Diameters of Arteriovenous Access and Factors Contributing to the Vessel Diameters on the Development of Complete Occlusive Access Thrombosis

**Figure 1:**
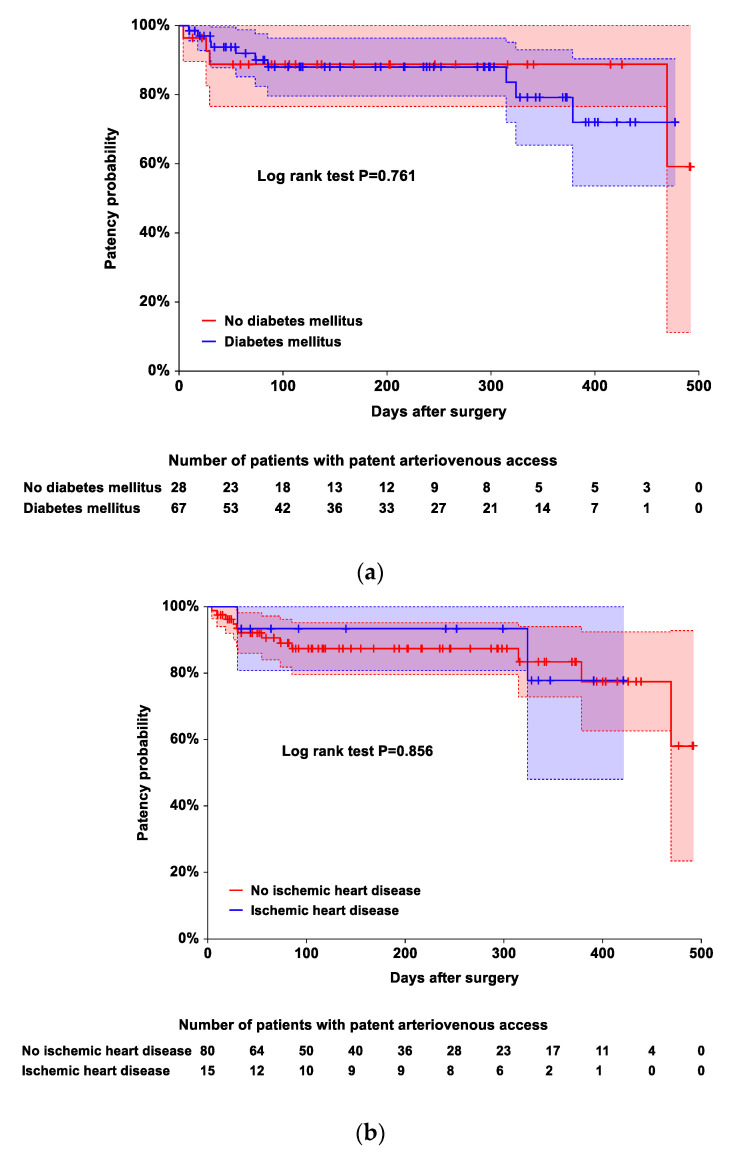

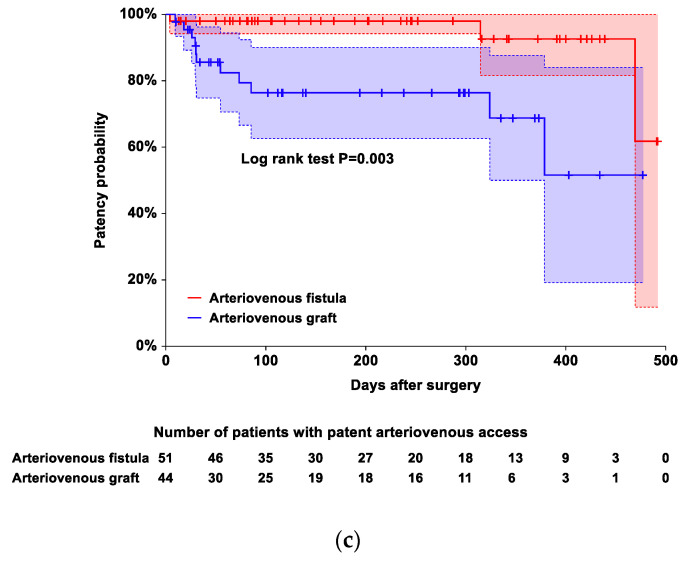
Kaplan–Meier plots showing the occurrence of complete occlusive access thrombosis during the follow-up periods according to the presence or absence of diabetes mellitus (**a**), presence or absence of ischemic heart disease (**b**), and arteriovenous access type (**c**). The time to the occurrence of complete occlusive access thrombosis was shorter in patients with arteriovenous grafts than in those with arteriovenous fistulas (*p* = 0.003 based on log-rank test). The area between the 2 dotted lines represents the 95% CIs of the Kaplan–Meier product-limit estimates. Crosses represent censored patients.

However, diabetes mellitus, ischemic heart disease, and post-BPB changes in the diameters of the inflow artery and outflow vein did not contribute to the risk of the occurrence of complete occlusive access thrombosis (Table 5 and Figure 1). The time to the occurrence of thrombosis was shorter in patients with AVGs than in those with AVFs (*p* = 0.003 based on log-rank test; hazard ratio: 5.748, 95% CI [1.584, 20.863], *p* = 0.008). The corresponding median survival time, restricted mean survival time, and restricted mean time lost are presented in Table 6.

## 4. Discussion

According to our results, diabetes mellitus and ischemic heart disease reduced the degree of increases in the diameters of the inflow artery and outflow veins, respectively. However, the two diseases and post-BPB changes in the diameters of the inflow artery and outflow vein did not have effects on the occurrence of complete occlusive access thrombosis. BPB consistently increased the diameters of the cephalic and axillary veins, while it had no effects on the diameter of the axillary artery in either group. In addition, BPB increased the diameters of both the inflow artery and outflow vein of the arteriovenous access, with more significant increases in the diameters of the outflow vein compared to those of the inflow artery. BPB increased the diameters of both the radial and brachial arteries in patients with a patent arteriovenous access, but their increases were not significant in patients with complete occlusive access thrombosis. The vasodilating effects of BPB benefited some patients because the original surgery plan was changed to a more favorable one. Despite the advantages of BPB in increasing vessel diameters, patients who received AVGs under BPB were still at risk of complete occlusive access thrombosis.

In vascular surgery, vasospasm is likely to occur because of increased sympathetic nervous activity and local manipulation of vessels. The resultant impairment of blood flow [14] leads to early thrombosis of the arteriovenous access. The dilation of the inflow artery and outflow veins accompanied by increases in their blood flow through the arteriovenous access in the perioperative period prevents thrombosis and early failure and promotes its maturation [5,15]. In this regard, BPB is preferable to local anesthesia [16,17]. Intraoperatively, the sympatholytic effects of BPB cause vasodilatation [4], a reduction in vascular resistance, and an increase in local blood flow [17]. The effects are maintained up to the early postoperative period [5,6]. In contrast, local anesthesia causes arterial and venous vasospasms [18] that reduce blood flow and cause local edema that increases the risk of infection [3] and reduces electrocautery efficiency. Furthermore, it provides insufficient anesthesia and necessitates systemic anesthetics [19] that pose a risk of respiratory depression in patients under hemodialysis [20]. In one randomized controlled trial involving 126 patients who were equally randomized to undergo BPB or local anesthesia for AVF creation, among the patients receiving a BPB, 16% of them had no presence of thrill or bruit 3 months after the surgery [7]. Although the incidence rate is significantly lower than that of patients undergoing local anesthesia (38%), it is still a concern that BPB cannot completely prevent the loss of patency. Similarly, in our study, 14.7% of the patients receiving a BPB developed complete occlusive access thrombosis (absence of thrill or bruit). Therefore, we investigated the factors that impede the benefits of BPB (vasodilation and the subsequent prevention of complete occlusive access thrombosis).

We found that diabetes mellitus and ischemic heart disease were associated with reductions in the extent of changes in both the inflow and outflow vascular diameters after BPB placement. Endothelial dysfunction is common in patients with diabetes [21] and ischemic heart disease [22]. In diabetic patients, high glucose levels delivered to endothelial progenitor cells originating from the bone marrow enhance the activity of nicotinamide adenine dinucleotide phosphate oxidase, which generates superoxide anion (O_2_^−^). O_2_^−^ inactivates nitric oxide, which is synthesized by endothelial nitric oxide synthase in vascular endothelial cells and relaxes vascular smooth muscles, causing vasodilation. In addition, O_2_^−^ uncouples endothelial nitric oxide synthase. Accordingly, nitric oxide production was found to be negatively correlated with plasma glucose and hemoglobin A1c levels in diabetic patients [23], and the peak systolic velocity of the dorsalis pedis artery was less increased after bilateral lumbar sympathicolysis in diabetic patients than in patients without diabetes mellitus [24]. Ischemic heart disease also induces oxidative stress where O_2_^−^ reduces nitric oxide concentration and endothelial nitric oxide synthase activity (uncoupling) [22]. Furthermore, a shift in the sympathovagal balance toward sympathetic predominance is associated with impaired endothelial function [25] in ischemic heart disease patients whose baseline sympathetic nervous activity is high [26]. Therefore, we speculate that nitric oxide-dependent vasoconstriction attenuated the vasodilating effects of BPB in patients with diabetes and ischemic heart disease.

Previous studies showed BPB-induced increases in the diameters of the forearm cephalic vein [27,28,29,30], upper arm cephalic vein [7,27,28,29,30], upper arm basilic vein [7,27,30], radial artery [7,28], brachial artery [7,28,29], and axillary vein [6]. Exceptionally, in one study, no significant change in the diameter of the forearm cephalic vein was observed [7]. Regrettably, in the abovementioned studies, the patterns of diameter changes between the patients with and without complete occlusive access thrombosis were not compared. Moreover, the vessel diameters were measured before the maximum vasodilating effects of BPB were achieved (immediately [7,30], 10 min [6,29], and unclear [27,28] after BPB placement), and we waited as long as we could (for 20 min after BPB placement) to measure vessel diameters under the near-maximum effects of BPB. In our study, unlike the patency group, the occlusion group did not show significant increases in the diameters of the radial and brachial arteries after the placement of the BPB. However, the nonsignificant diameter changes did not seem to contribute to the occurrence of complete occlusive access thrombosis because we did not use the arteries, the diameters of which were not increased by BPB enough to meet the criteria for arteriovenous access surgery. Accordingly, the diameters of the inflow artery used for the arteriovenous access were significantly increased by BPB in the occlusion group as in the patency group. In particular, in our study, the changes in the diameters of the outflow veins were more pronounced after the placement of BPB than the changes in the diameters of the inflow arteries. Likewise, one prospective observational study showed results similar to ours [28]. However, in another randomized, controlled study, the changes in vessel diameters after the placement of BPB were not consistent between arteries and veins [7]. We speculate that the inconsistent results between the studies are attributed to the differences in vessel diameter measurement time after BPB and the dose/type of local anesthetic used for BPB.

In previous studies, researchers reported the superiority of AVF to AVG regarding their postoperative patency [31,32]. However, the researchers did not consider the effects of anesthesia type on the surgical outcome. Even though we performed a BPB that dilated the blood vessels to provide favorable surgical conditions for arteriovenous access creation, AVG was still inferior to AVF, which was consistent with the results of previous studies.

Some limitations should be considered regarding this study. Because of the retrospective design of this study, an association rather than a cause-and-effect relationship between variables was allowed in the investigation. In addition, based on our results, it is unclear why diabetes and ischemic heart disease had different effects on the diameters of the inflow artery and the outflow vein of the arteriovenous access even though BPB led to dilations in both the artery and vein of the upper limb [6,7,27,28,29,30]. Last, because of the low number (n = 14) of patients with complete occlusive access thrombosis, multiple Cox proportional hazard regression could not be used to assess the combined effects of independent variables on the occurrence of complete occlusive access thrombosis [13].

## 5. Conclusions

In summary, although BPB did not increase the diameters of the arteries in the occlusion group compared to the patency group, it led to more substantial dilations of the outflow veins than the inflow arteries in both groups. Diabetes and ischemic heart disease attenuate the vasodilating effects of BPB in the inflow and outflow vessels, respectively. However, the two diseases do not have effects on the occurrence of complete occlusive access thrombosis. In particular, the vasodilating effects of BPB did not improve the surgical outcome of patients with AVGs. Therefore, further studies are warranted to investigate the factors contributing to surgical outcomes in patients undergoing arteriovenous access surgery under BPB.

## Figures and Tables

**Table 1:** Patient characteristics.

	Patency Group (n = 81)	Occlusion Group (n = 14)	Mean or Median Difference (95% CI)	*p* Value
Age (yr.)	66 (53, 75)	59 (49, 71)	3 (−4, 10)	0.446
Sex (M/F)	47 (58.0)/34 (42.0)	8 (57.1)/6 (42.9)	Not applicable	0.951
Height (cm)	161.4 ± 9.3	164.1 ± 2.8	−2.8 (−8.2, 2.7)	0.314
Weight (kg)	59.8 ± 11.7	60.1 ± 11.2	−0.4 (−7.0, 6.3)	0.916
Body mass index (kg/m^2^)	22.9 ± 3.6	22.3 ± 3.2	0.6 (−1.4, 2.7)	0.552
Smoking history	12 (14.8)	4 (28.6)	Not applicable	0.245
Underlying disease				
Diabetes mellitus	57 (70.4)	10 (71.4)	Not applicable	1.000
Hypertension	59 (72.9)	12 (85.7)	Not applicable	0.506
Ischemic heart disease	13 (16.1)	2 (14.3)	Not applicable	1.000
Cerebrovascular accident	19 (23.5)	2 (14.3)	Not applicable	0.728
Hyperlipidemia	4 (5.0)	2 (14.3)	Not applicable	0.214
Pulmonary disease	1 (1.2)	0 (0.0)	Not applicable	1.000
Miscellaneous	13 (16.1)	1 (7.1)	Not applicable	0.685
Side of surgery (right/left)	12 (14.8)/69 (85.2)	4 (28.6)/10 (71.4)	Not applicable	0.245
Type of BPB				0.833
Supraclavicular	67 (82.7)	11 (78.6)	Not applicable	
Axillary	11 (13.6)	2 (14.3)	Not applicable	
Infraclavicular	3 (3.7)	1 (7.1)	Not applicable	
Block performance time (min)	5 (4, 6)	5 (3.8, 5.3)	0 (−1, 1)	0.446
Volume of 1.5% mepivacaine used for BPB (ml)	25 (23, 31.5)	28.5 (23.8, 32.8)	−1 (−5, 2)	0.482
Surgical procedure				<0.001
Brachial-cephalic fistula	40 (49.4)	1 (7.1)	Not applicable	
Brachial-axillary bridge graft	25 (30.9)	6 (42.9)	Not applicable	
Radial-cephalic fistula	8 (9.9)	2 (14.3)	Not applicable	
Brachial-antecubital forearm loop graft	7 (8.6)	1 (7.1)	Not applicable	
Brachial-basilic forearm loop graft	1 (1.2)	3 (21.4)	Not applicable	
Axillo-axillary loop graft	0 (0.0)	1 (7.1)	Not applicable	
AVF/AVG	48 (59.3)/33 (40.7)	3 (21.4)/11 (78.6)	Not applicable	0.009
Change in original operation plan	7 (8.6)	0 (0.0)	Not applicable	0.589
Duration of surgery (min)	65 (58, 83)	83 (62, 114)	−12 (−29, 1)	0.071
Duration of anesthesia (min)	94 (81.5, 108.5)	113.5 (86.3, 142.8)	−15 (−33, 1)	0.064
Follow-up duration (days)	202 (81.5, 338)	43 (24, 317.3)	73.5 (12, 180)	0.018

Values are mean ± SD, median (1st quartile, 3rd quartile), or number of patients (percentage). AVF: arteriovenous fistula; AVG: arteriovenous graft; BPB: brachial plexus block.

**Table 2:** Vessel diameters measured before and after brachial plexus block.

	Overall (n = 95)	Patency Group (n = 81)	Occlusion Group (n = 14)	Median Difference (95% CI) (Patency Group vs. Occlusion Group)	*p* Value
Radial artery					
Before BPB (mm)	2.2 (1.8, 2.6)	2.2 (2, 2.55)	1.75 (1.5, 2.6)	0.3 (−0.1, 0.7)	0.136
After BPB (mm)	2.3 (2, 2.7) *	2.3 (2, 2.7) *	2 (1.73, 2.53)	0.2 (0, 0.6)	0.077
Change between before and after BPB (mm)	0 (0, 0.2)	0.1 (0, 0.2)	0 (−0.13, 0.28)	0 (−0.1, 0.2)	0.685
Brachial artery					
Before BPB (mm)	4.4 (3.9, 4.8)	4.3 (3.9, 4.8)	4.7 (3.85, 5.05)	−0.2 (−0.7, 0.2)	0.394
After BPB (mm)	4.5 (4, 5) *	4.5 (4, 4.95) ^†^	4.75 (4.3, 5.4)	−0.3 (−0.7, 0.2)	0.267
Change between before and after BPB (mm)	0.1 (−0.1, 0.3)	0.1 (−0.1, 0.25)	0.2 (−0.05, 0.4)	−0.1 (−0.2, 0.1)	0.349
Axillary artery					
Before BPB (mm)	5.5 (5, 6.4)	5.6 (5, 6.4)	5.15 (4.7, 6.48)	0.4 (−0.2, 0.9)	0.167
After BPB (mm)	5.6 (5, 6.4)	5.6 (5, 6.35)	5.7 (4.6, 6.5)	0.2 (−0.4, 0.7)	0.603
Change between before and after BPB (mm)	0 (−0.3, 0.2)	0 (−0.3, 0.2)	0.15 (−0.1 0.3)	−0.1 (−0.3, 0.1)	0.292
Forearm cephalic vein					
Before BPB (mm)	1.5 (1.3, 1.9)	1.6 (1.3, 1.95)	1.45 (1.2, 1.8)	0.1 (−0.1, 0.3)	0.317
After BPB (mm)	1.7 (1.4, 2.2) *	1.7 (1.5, 2.2) *	1.65 (1.3, 2.03) ^‡^	0.1 (−0.3, 0.3)	0.624
Change between before and after BPB (mm)	0.1 (0, 0.3)	0.1 (0, 0.35)	0.2 (0, 0.33)	−0.1 (−0.2, 0.1)	0.459
Upper arm cephalic vein					
Before BPB (mm)	2.8 (2.4, 3.5)	2.7 (2.4, 3.5)	3.15 (2.3, 3.65)	−0.1 (−0.7, 0.4)	0.647
After BPB (mm)	3.4 (2.8, 4) *	3.4 (2.8, 4) *	3.05 (2.7, 4.35) ^†^	0.1 (−0.4, 0.6)	0.694
Change between before and after BPB (mm)	0.5 (0.2, 0.8)	0.5 (0.2, 1)	0.4 (0.28, 0.73)	0.1 (−0.1, 0.4)	0.307
Axillary vein					
Before BPB (mm)	6.3 (5.1, 7.1)	6.3 (5.15, 7.2)	6.05 (4.88, 6.83)	0.2 (−0.4, 1)	0.481
After BPB (mm)	6.8 (5.8, 7.5) *	6.8 (5.9, 7.6) *	6.45 (5.38, 7.1) ^‡^	0.4 (−0.3, 1.1)	0.213
Change between before and after BPB (mm)	0.4 (0.1, 1.1)	0.4 (0.1, 1.1)	0.45 (0.1, 1.03)	0 (−0.4, 0.4)	0.916
Inflow artery used for the arteriovenous access					
Before BPB (mm)	4.3 (3.8, 4.8)	4.2 (3.8, 4.8)	4.55 (3.6, 4.93)	−0.1 (−0.6, 0.5)	0.789
After BPB (mm)	4.4 (3.8, 4.9) ^‡^	4.4 (3.8, 4.9) ^‡^	4.4 (3.93, 5.1) ^‡^	−0.1 (−0.6, 0.4)	0.632
Change between before and after BPB (mm)	0.1 (−0.1, 0.2)	0.1 (−0.1, 0.2)	0.15 (0, 0.4)	−0.1 (−0.2, 0.1)	0.297
Outflow vein used for the arteriovenous access					
Before BPB (mm)	3.5 (2.5, 5.3)	3.4 (2.5, 5.3)	4.3 (2.88, 5.98)	−0.5 (−1.6, 0.6)	0.318
After BPB (mm)	4.2 (3.2, 5.7) *	4 (3.1, 5.7) *	4.8 (3.35, 6.38) ^†^	−0.4 (−1.4, 0.6)	0.386
Change between before and after BPB (mm)	0.6 (0.3, 1) ^§^	0.6 (0.3, 1.05) ^§^	0.6 (0.3, 1.03) ^‖^	0 (−0.3, 0.4)	0.870

Values are median (1st quartile, 3rd quartile). * *p* < 0.001, ^†^ *p* < 0.01, and ^‡^ *p* < 0.05 compared to before BPB. ^§^ *p* < 0.001 and ^‖^ *p* < 0.05 compared to inflow artery AVF. AVF: arteriovenous fistula; AVG: arteriovenous graft; BPB: brachial plexus block.

**Table 3:** Multiple linear regression analysis of the predicted changes in the diameter of the inflow artery from the arteriovenous access after brachial plexus block.

Independent Variable	Partial Regression Coefficient (95% CI)	*p* Value
Age	0.002 (−0.001, 0.006)	0.223
Smoking history	0.137 (−0.002, 0.276)	0.054
Diabetes mellitus	−0.183 (−0.301, −0.065)	0.003
Ischemic heart disease	0.123 (−0.022, 0.268)	0.096
Cerebrovascular accident	0.081 (−0.044, 0.206)	0.200

Coefficient of determination (R^2^) = 0.141, adjusted R^2^ = 0.093, *p* = 0.017. AVF: arteriovenous fistula; AVG: arteriovenous graft.

**Table 4:** Multiple linear regression analysis of the predicted changes in the diameter of the outflow vein from the arteriovenous access after brachial plexus block.

Independent Variable	Partial Regression Coefficient (95% CI)	*p* Value
Age	0.008 (−0.002, 0.017)	0.100
Smoking history	0.018 (−0.345, 0.380)	0.923
Diabetes mellitus	−0.285 (−0.592, 0.023)	0.068
Ischemic heart disease	−0.402 (−0.781, −0.024)	0.038
Cerebrovascular accident	−0.248 (−0.573, 0.077)	0.133

Coefficient of determination (R^2^) = 0.124, adjusted R^2^ = 0.074, *p* = 0.036. AVF: arteriovenous fistula; AVG: arteriovenous graft.

**Table 5:** Univariate Cox proportional hazard regression analysis to predict the risk of the occurrence of complete occlusive access thrombosis at a specific time.

Independent Variable	Hazard Ratio (95% CI)	*p* Value
Age	1.001 (0.964, 1.040)	0.956
Diabetes mellitus	1.205 (0.362, 4.009)	0.761
Ischemic heart disease	0.869 (0.192, 3.934)	0.856
Change in diameter of inflow artery	3.563 (0.340, 37.378)	0.289
Change in diameter of outflow vein	0.833 (0.362, 1.916)	0.667
Arteriovenous graft	5.748 (1.584, 20.863)	0.008

**Table 6:** Kaplan–Meier survival analysis.

**Diabetes Mellitus**	**Absent (n = 28)**	**Present (n = 67)**
Median occlusion-free time (days [95% CI])	Not applicable	Not applicable
Restricted mean occlusion-free time (days [95% CI])	423.1 (368.2, 477.9)	404.5 (364.2, 444.9)
Restricted mean time lost (days [95% CI])	53.9 (−0.9, 108.8)	72.5 (32.1, 112.8)
**Ischemic Heart Disease**	**Absent (n = 80)**	**Present (n = 15)**
Median occlusion-free time (days [95% CI])	Not applicable	Not applicable
Restricted mean occlusion-free time (days [95% CI])	366.2 (335.8, 396.5)	379.8 (325.4, 434.3)
Restricted mean time lost (days [95% CI])	54.8 (24.5, 85.2)	41.2 (−13.3, 95.6)
**Arteriovenous Access Type**	**AVF (n = 51)**	**AVG (n = 44)**
Median occlusion-free time (days [95% CI])	Not applicable	Not applicable
Restricted mean occlusion-free time (days [95% CI])	456.4 (432.0, 480.9)	345.8 (281.9, 409.7)
Restricted mean time lost (days [95% CI])	20.6 (−3.9, 45.0)	131.2 (67.3, 195.1)

AVF: arteriovenous fistula; AVG: arteriovenous graft.

## Data Availability

The data presented in this study are available on request from the corresponding author.

## Supplementary Materials

The following supporting information can be downloaded at: https://www.mdpi.com/article/10.3390/ijerph192215158/s1. Table S1. Statistical analysis.
