# Supplementary material for: The Effects of Vasodilation Induced by Brachial Plexus Block on the Development of Postoperative Thrombosis of the Arteriovenous Access in Patients with End-Stage Renal Disease: A Retrospective Study
Source: Int J Environ Res Public Health. 2022 Nov 17;19(22):15158. doi: 10.3390/ijerph192215158 (PMC9690458; doi:10.3390/ijerph192215158)
Supplement: Supplementary file 1 [file ijerph-19-15158-s001.zip › ijerph-1930842-supplementary.pdf]

Table S1. Statistical analysis

Statistical method	Details
Shapiro-Wilk test	• Tests the normality assumption.
Independent 2-sample Student's t test	• Compares a normally distributed continuous variable between 2 groups
Mann-Whitney U test	• Compares a non-normally distributed variable between 2 groups
Hodges-Lehmann estimator	• Calculates a median difference between 2 groups and its 95% CI
Chi-square test	• Compares categorical data with more than 1 level between 2 groups
Fisher's exact test	• Compares categorical data with 2 levels between 2 groups if the expected frequency of any cell is less than 5.
Wilcoxon signed rank test	• Compares 2 non-normally distributed paired variables within a group.<br>• Assesses the longitudinal changes in vessel diameters from before to after BPB and the within-group difference between vessel diameter changes in both the inflow artery and outflow vein of the arteriovenous access after BPB.
Multiple linear regression	• Assesses the linear relationships between more than one independent variable and one dependent variable.<br>• The independent variables included in the multiple linear regression analysis were age, sex, body mass index, BPB type, the volume of mepivacaine used for BPB, smoking history, and underlying diseases.<br>• Independent variables with a variance inflation factor >5 were regarded as multicollinear with some of the other variables [1].<br>• Independent variables, the variance decomposition proportions of which were >0.3 at the common condition index (>10), were regarded as multicollinear with one another [1,2].<br>• Among the multicollinear variables, more clinically irrelevant variables were excluded first from the regression models.<br>• The final multiple linear regression models had differences <0.1 between the coefficient of determination (R^2) and the adjusted R^2.
Univariate Cox proportional hazards regression	• Predicts the occurrence of complete occlusive access thrombosis at a specific time with one independent variable.<br>• The independent variables included were age, diabetes mellitus, ischemic heart disease, post-BPB changes in the diameters of the inflow artery and the outflow vein from the arteriovenous access, and arteriovenous access type [AVG vs. AVF creation]).<br>• Because 10 events per independent variable are required to obtain an unbiased multiple Cox proportional hazards regression model [3], univariate regression analysis was performed based on the number of patients developing complete occlusive access thrombosis, which was 14.
Kaplan-Meier survival analysis	• Calculates event-free probabilities between the beginning and end of the follow-ups.<br>• A log-rank test was used to compare the Kaplan-Meier survival plots between 2 groups.

- The survival plots were compared between patients with and without diabetes mellitus, between patients with and without ischemic heart disease, and between AVF and AVG.
- The median survival time, restricted mean survival time, and restricted mean time lost were calculated from the survival functions.

AVF: arteriovenous fistula, AVG: arteriovenous graft, BPB: brachial plexus block.

References

1. Kim, J.H. Multicollinearity and misleading statistical results. *Korean J Anesthesiol* **2019**, *72*, 558-569, doi:10.4097/kja.19087.
2. Weissfeld, L.A. A multicollinearity diagnostic for models fit to censored data. *Communications in Statistics-Theory and Methods* **1989**, *18*, 2073-2085.
3. Peduzzi, P.; Concato, J.; Feinstein, A.R.; Holford, T.R. Importance of events per independent variable in proportional hazards regression analysis. II. Accuracy and precision of regression estimates. *J Clin Epidemiol* **1995**, *48*, 1503-1510, doi:10.1016/0895-4356(95)00048-8.
